# Biweekly rituximab, cyclophosphamide, vincristine, non-pegylated liposome-encapsulated doxorubicin and prednisone (R-COMP-14) in elderly patients with poor-risk diffuse large B-cell lymphoma and moderate to high ‘life threat’ impact cardiopathy

**DOI:** 10.1111/j.1365-2141.2011.08786.x

**Published:** 2011-09

**Authors:** Gaetano Corazzelli, Ferdinando Frigeri, Manuela Arcamone, Anna Lucania, Maria RosariaVilla, Emanuela Morelli, Alfonso Amore, Gaetana Capobianco, Antonietta Caronna, Cristina Becchimanzi, Francesco Volzone, Gianpaolo Marcacci, Filippo Russo, Rosaria De Filippi, Lucia Mastrullo, Antonio Pinto

**Affiliations:** 1 Haematology-Oncology and Stem Cell Transplantation Unit, National Cancer InstituteFondazione ‘G. Pascale’, IRCCS; 2 HaematologyA.O. San Gennaro, ASL NA1; 3 EGBP Surgery, Department of Abdominal Surgery D, National Cancer InstituteFondazione ‘G. Pascale’, IRCCS; 4 Cardiology, National Cancer Institute, Fondazione ‘G. Pascale’IRCCS; 5 Department of Cellular and Molecular Biology and Pathology, Faculty of Biotechnological Sciences, Federico II UniversityNaples, Italy

**Keywords:** non-pegylated liposomal doxorubicin, elderly, diffuse large B-cell lymphoma, cardiotoxicity, Charlson Comorbidity Index

## Abstract

This Phase II study assessed feasibility and efficacy of a biweekly R-COMP-14 regimen (rituximab, cyclophosphamide, non-pegylated liposome-encapsulated doxorubicin, vincristine and prednisone) in untreated elderly patients with poor-risk diffuse large B-cell lymphoma (DLBCL) and moderate to high ‘life threat’ impact NIA/NCI cardiac comorbidity. A total of 208 courses were delivered, with close cardiac monitoring, to 41 patients (median age: 73 years, range: 62–82; 37% >75 years) at a median interval of 15·6 (range, 13–29) days; 67% completed all six scheduled courses. Response rate was 73%, with 68% complete responses (CR); 4-year disease-free survival (DFS) and time to treatment failure (TTF) were 72% and 49%, respectively. Failures were due to early death (*n* = 3), therapy discontinuations (no-response *n* = 2; toxicity *n* = 6), relapse (*n* = 6) and death in CR (*n* = 3). Incidence of cardiac grade 3–5 adverse events was 7/41 (17%; 95% confidence interval: 8–31%). Time to progression and overall survival at 4-years were 77% and 67%, respectively. The Age-adjusted Charlson Comorbidity Index (aaCCI) correlated with failures (*P* = 0·007) with patients scoring ≤7 having a longer TTF (66% vs. 29%; *P* = 0·009). R-COMP-14 is feasible and ensures a substantial DFS to poor-risk DLBCL patients who would have been denied anthracycline-based treatment due to cardiac morbidity. The aaCCI predicted both treatment discontinuation rate and TTF.

Elderly patients with newly diagnosed diffuse large B-cell lymphoma (DLBCL) and a poor prognostic profile, according to the International Prognostic Index (IPI), were shown to benefit from shortening of the time interval between CHOP (cyclophosphamide, doxorubicin, vincristine and prednisone) chemotherapy cycles from 3 weeks (CHOP-21) to 2 weeks (CHOP-14) (Pfreundschuh *et al*, 2004, 2008). In these patients, biweekly CHOP plus rituximab (R) (R-CHOP-14) is regarded as a therapeutic standard for some cooperative groups and is a valuable option in clinical practice (Pfreundschuh *et al*, 2008; Rueda *et al*, 2008; Ziepert *et al*, 2010; Zwick *et al*, 2007).

Further exploration of dose-densified approaches to treat high-risk DLBCL in the elderly appears also justified due to: (i) the high initial relapse rate in elderly patients (60–80 years old) with poor IPI risk given R-CHOP-21 (Feugier *et al*, 2005) and their low chance of achieving a durable second remission (Corazzelli *et al*, 2009; Gisselbrecht *et al*, 2009), (ii) the comparable toxicity of R-CHOP-21 and R-CHOP-14 in terms of cardiac complications (Cunningham *et al*, 2009; Delarue *et al*, 2009; Pfreundschuh *et al*, 2004), (iii) the prospect of a less protracted, if not halved, therapy duration in elderly patients who display a limited physical and psychosocial compliance to prolonged treatments, and (iv) the recent recognition of the highly aggressive entity ‘Epstein-Barr virus (EBV)-positive DLBCL of the elderly’ (12% to 33% of DLBCL) with a median progression free survival of only about 1 year upon CHOP-21 +/− Rituximab (Park *et al*, 2007; Swerdlow *et al*, 2008).

Delivery of densified R-CHOP in older patients with heart-related morbidities, may however, be challenging because of the acute, early and late cardiotoxicities of doxorubicin (Grann *et al*, 2006; Hershman *et al*, 2008). Prolongation of the QTc interval, arrhythmias, myopericarditis and increase of N-terminal (NT)-pro brain natriuretic peptide (proBNP), an early biomarker of ventricular dysfunction, were described during CHOP-like programmes, independently of the cumulative doxorubicin dosing (Elliott, 2006; Johnson, 2006). In general, the suggestion that fit patients with DLBCL, even if aged more than 80 years, should be evaluated for ‘adapted’ anthracycline-based regimens with curative intent (Bairey *et al*, 2006; Peyrade *et al*, 2010; Thieblemont *et al*, 2008) has to face the problem of cardiac risk factors and comorbidities. In this regard, the therapeutic approach remains pragmatic also due to the absence of international guidelines on cardioprotective agents and of uniform strategies for patients with cardiac risk factors (Hensley *et al*, 2009). A consensus regarding the optimal methods for cardiac monitoring during anthracycline chemotherapy is also lacking (Altena *et al*, 2009), so that current guiding principles are about the same as those used 20 years ago (Cheitlin *et al*, 2003; Klocke *et al*, 2003; Schwartz *et al*, 1987). Such issues are very relevant in the context of population-based practice, i.e. involving ‘non-study’ patients, where, differently from controlled trials, selection criteria and therapy optimization do not usually apply (Thieblemont & Coiffier, 2007).

The application of liposome-encapsulated formulations of doxorubicin has been suggested as a strategy to minimize cardiac side effects and favour a more selective drug uptake by lymphoma cells (Allen & Martin, 2004). Liposomal formulations display comparable efficacy with free anthracyclines, but are linked to more favourable bioavailability and biodistribution profiles (Batist *et al*, 2001; Levine *et al*, 2004; Tsavaris *et al*, 2002; Zaja *et al*, 2006). This enables the delivery of higher cumulative doses of liposomal anthracyclines with a lower risk of heart failure as compared to the parent compounds (Young *et al*, 2004). Whether the use of these liposome-encapsulated formulations might also represent a tool for the application of curative dose-intense regimens to older patients with highly chemosensitive tumours in the presence of cardiac risk factors, remains to be assessed.

To this end, we have conducted a pilot study to explore a dose-dense R-CHOP-like regimen (R-COMP-14) including non-pegylated liposome-encapsulated doxorubicin (NPLD) in elderly DLBCL patients falling into a poor-prognostic group by standard IPI and presenting moderate to high ‘life threat’ impact cardiopathy, as defined by the National Institute on Aging and the National Cancer Institute (NIA/NCI) criteria applied to non-Hodgkin lymphoma (NHL) (Janssen-Heijnen *et al*, 2005; Yancik *et al*, 1998). Patients were concurrently assessed through the age-adjusted Charlson Comorbidity Index (aaCCI) (Charlson *et al*, 1987; Hall *et al*, 2004; Thieblemont & Coiffier, 2007). We evaluated the feasibility of this biweekly R-COMP regimen, as indicated by the actual proportion of patients fully receiving the planned treatment, the incidence of cardiac adverse events (AE), and its therapeutic efficacy in terms of response and survival outcomes.

## Patients and methods

### Patients selection

This unsponsored study was approved by local ethic committees and conducted according to the Good Clinical Practice guidelines and the Declaration of Helsinki. All patients gave written informed consent to treatment and use of clinical data for research. Eligible patients were required to have newly diagnosed histologically confirmed DLBCL according to the World Health Organization (WHO) Lymphoma Classification (Jaffe *et al*, 2001), age > 60 years, intermediate-high and high standard IPI risk (The International Non-Hodgkin’s Lymphoma Prognostic Factors Project., 1993) and one or more condition(s) of moderate to high ‘life threat’ impact cardiac morbidity. This latter was defined according to the NIA/NCI index (Yancik *et al*, 1998) as applied to non-Hodgkin lymphoma patients (Janssen-Heijnen *et al*, 2005). In this regimen, an equal dose of NLPD was substituted for doxorubicin within the R-CHOP-14 platform. The following eligibility criteria were also required: negativity for human immunodeficiency virus and surface antigen of hepatitis B, Eastern Cooperative Group performance status (ECOG PS) 0–3, no previous chemotherapy or radiotherapy, creatinine clearance (CrCl) >30 ml/min, serum transaminases less than three times the normal value, bilirubin <34·2 μmol/l, absolute neutrophil count ≥1000 × 10^9^/l, haemoglobin level ≥100 g/l, and platelet count ≥75 000 × 10^9^/l, no history of congestive heart failure (CHF), left ventricular ejection fraction (LVEF) ≥45% at bi-dimensional echocardiography or, in selected cases (i.e. obese patient), multiple uptake gated acquisition (MUGA) scintigraphy. Myocardial infarction represented an exclusion criteria only if diagnosed within 12 months prior to R-COMP-14.

Comorbidity was assessed with the aaCCI (Charlson *et al*, 1987; Hall *et al*, 2004). Pre-treatment disease assessment included a contrast-enhanced total-body computed tomography scan, Fluorine-18 (18F)-Fluorodeoxyglucose Positron Emission Tomography (FDG-PET), bone marrow (BM) biopsy and lumbar puncture in case of testicular, breast, epidural or sinus involvement. Restaging was scheduled after three courses and a final evaluation was performed 4 weeks after the end of chemotherapy. No consolidation radiotherapy was scheduled.

### Treatment

Patients were scheduled to receive six courses of R-COMP-14 (rituximab 375 mg/m^2^ on day 1, cyclophosphamide 750 mg/m^2^ on day 2, NLPD-Myocet® 50 mg/m^2^ on day 2, vincristine 1·4 mg/m^2^, up to a maximal dose of 2 mg on day 2, and prednisone 40 mg/m^2^ per day for 5 d) at a 2-week interval. Myocet® was purchased from Cephalon, Rome, Italy. Primary granulocyte colony-stimulating factor (G-CSF) support was employed from day 6 to day 10 of each course. The agent-specific relative dose intensity (RDI) for each drug was calculated as the ratio between the administered dose and the planned dose, described in mg/m^2^ per week. The regimen was administered on an inpatient basis for the first four courses and a 3–5 d prednisone pretreatment was given before the first cycle to patients with ECOG PS >1. All patients received rasburicase and appropriate hydration/alkalinization during the first course. Intrathecal prophylaxis with 50 mg of liposome-encapsulated cytarabine (Depocyte ®; Mundipharma, Cambridge, UK), on day 2 of cycles 1–4, was given in cases at risk for central nervous system (CNS) localization. No continuous use of acyclovir and cothrimoxazole was recommended but patients received ciprofloxacin and/or azythromycin prophylaxis, while fluconazole was employed as needed; febrile neutropenia was managed by hospitalization.

The prescription of QTc interval-prolonging drugs, such as some fluoroquinolones, macrolide antibiotics and itraconazole, was discouraged (Liu & Juurlink, 2004; Wolbrette, 2004), as well as use of tricyclic antidepressants, haloperidol and selective serotonin reuptake inhibitors (Liu & Juurlink, 2004; Wolbrette, 2004). Electrocardiography was repeated before each course together with bi-dimensional echocardiographic survey. No protocol-committed pre-emptive strategy was planned with agents recognized to have protective activity from doxorubicin-related cardiotoxicity, such as beta-blockers, angiotensin-converting-enzyme inhibitors (ACEI) and angiotensin receptor blockers (ARB); however patients continued to take their daily background cardiological medications, including beta-blockers and ACEI/ARB if previously prescribed.

### Toxicity and dose modulation

Toxicity was evaluated using the NCI Common Terminology Criteria for Adverse Events (CTCAE) v3.0 toxicity scale (http://ctep.cancer.gov/protocolDevelopment/electronic_applications/docs/ctcaev3.pdf). Full blood cell counts, together with renal and hepatic function tests, were planned twice weekly after the first R-COMP-14 and weekly thereafter. The next chemotherapy cycle was scheduled at day 15, if neutrophil count >1000 × 10^9^/l and platelet count >75 000 × 10^9^/l. If the platelet count was <75 000 × 10^9^/l the course was delayed for up to 2 weeks. In case of grade 4 thrombocytopenia, the doses of cyclophosphamide and doxorubicin were decreased by 25%. Dose reduction guidelines also included: (i) vincristine reduction, to a fixed dose of 1 mg, in case of grade 2 peripheral neuropathy or serum bilirubin 25·65–51·3 μmol/l or transaminases 2–3 times upper limit of normal (ULN) or alkaline phosphatase increased; decrease to 25% of dose if serum bilirubin 51·3–85·5 μmol/l; avoid use for grade 3–4 peripheral neuropathy, or serum bilirubin >85·5 μmol/l or aspartate transaminase >180 units, (ii) decrease cyclophosphamide to 75% of dose in case of CrCl <10 ml/min; and serum bilirubin 53–85·5 μmol/l or transaminases >3 times ULN; avoid use if serum bilirubin >85·5 μmol/l, and (iii) decrease of NPLD to 50% for bilirubin 34·2–51·3 μmol/l; to 25% for bilirubin 51·3–85·5 μmol/l. The doses of rituximab were not modified. Treatment was stopped in case of no response, lymphoma progression, patient refusal or, at the treating physicians judgment, in cases of intercurrent illness. Therapy was also withdrawn in case of the following CTCAE v3.0 grade 3 and 4 cardiac adverse events: (i) conduction abnormality/arrhythmia incompletely controlled medically (grade 3) or associated with CHF, hypotension, syncope or shock (grade 4), (ii) symptoms and testing consistent with ischaemia, unstable angina (grade 3) or acute myocardial infarction (grade 4), (iii) cardiac troponin serum levels consistent with unstable angina (grade 3) or myocardial infarction (grade 4), (iv) symptomatic left ventricular systolic dysfunction with CHF, or resting ejection fraction <40% responsive to intervention (grade 3) or poorly controlled (grade 4), (v) pericardial effusion with physiological (grade 3) or life-threatening consequences (grade 4), and (vi) symptomatic valvular disease controlled with medical therapy (grade 3), or life-threatening/disabling (grade 4).

### Endpoints

The primary safety endpoint was the incidence of cardiac events, defined as definite cardiac death, sudden death without documented non-cardiac aetiology and the occurrence of grade 3 and 4 cardiac adverse events according to CTCAE v3.0. The primary efficacy endpoints were complete response (CR) rate and time to treatment failure (TTF). Criteria for remission and survival outcomes were as published (Cheson *et al*, 2007). TTF was computed from the first day of treatment to either disease progression, relapse, or R-COMP-14 discontinuation due to patient refusal, grade 3 and 4 cardiac AE or the treating physicians decision on intercurrent illness. Death without progression within 4 weeks after the start of therapy was designated as early therapy-related death. Death without progression after completion of the whole treatment was also considered therapy-related if due to cardiac events. The age cut-off of 70 years and aaCCI were analysed for correlation with TTF.

Secondary endpoints were time to progression (TTP), overall survival (OS) and disease-free survival (DFS) in complete responders. TTP was measured from the first day of treatment to the date of documented lymphoma progression or death as a result of lymphoma. OS was measured from the first day of treatment to the date of death as a result of any cause or last follow-up. DFS was calculated from the time of occurrence of CR until relapse or death as a result of lymphoma or treatment toxicity.

### Statistics

In order to avoid unacceptable toxicity, the Bryant and Day two-stage phase II trial design was chosen with α_R_ = α_T_ = 0·1 and β = 0·2. A cut-off point for the response rate (70%) and for severe cardiac events (20%) was established for the first 19 patients. On this basis, the completed stage-one trial of 19 patients proceeded to stage-two, up to 40 patients, if there were at least 11 responses and 13 ‘safe’ patients (Bryant & Day, 1995). Probabilities of early termination of the study in case of excessive toxicity were 72% in the presence of good response and 90% in case of poor response. Survival endpoints were analysed using the approach of Kaplan and Meier and estimations at 4 years were calculated with 95% confidence intervals (CI). Univariate analyses were performed using Fisher’s exact test and log rank test. Non-parametric linear regression was performed for correlation analysis. All P values were two-tailed. All efficacy and toxicity endpoints were updated at December 2010. Statistical analysis was performed with the Statistical Package for the Social Sciences (spss), version 14.0 (SPSS, Chicago, IL, USA).

## Results

A total of 41 patients were prospectively accrued between January 2007 and July 2009; the two stages of the study design were both completed in the absence of excessive toxicity or unacceptably poor outcome and all patients were evaluable for toxicity and response. In stage-one of the study 19 patients were enrolled: the overall response rate (ORR) was 73·7% [13 CRs and one partial response (PR)], with severe cardiac events occurring in 11% of patients. Given that a response rate of at least 70% and a ≤20% incidence of severe cardiac events were required to proceed to stage-two, an additional 22 patients were enrolled. No statistically significant differences were detected between patients accrued in the two stages in terms of demographics, disease characteristics, comorbidity at presentation, and response to treatment (Table S1).

### Base-line characteristics

The clinical features of the entire patient population are summarized in Table I. The median age at the time of inclusion was 73 years and 15 patients (37%) were older than 75 years. Twenty-four patients had an IPI of 3, and in 17 the IPI was ≥4; 27 patients (71%) were in stage IV, 49% had B symptoms and 32% were assigned an ECOG PS > 1.

**Table I:** Patient characteristics

Characteristics	*n*	%
Patients entered	41	100
Age at treatment (years)		
Median (range)	73 (62–82)	
61–65	6	15
66–70	9	22
71–75	11	27
76–80	10	24
>80	5	12
Male gender	23	56
Clinical stage		
II	4	
III	10	24
IV	27	66
B symptoms	20	49
No of extranodal sites ≥ 2	14	34
Bone marrow involvement	9	22
Lactate dehydrogenase > ULN	33	81
ECOG performance Status ≥ 2	13	32
Standard IPI score		
3	24	59
4–5	17	41
Basal LVEF value (%)		
Median	57	
Range	45–67	
Cardiovascular risk factors		
Chronic renal failure (GFR 30–50 ml/min per 1·73 m^2^)	5	12
Hypertension, UAT	12	
Diabetes mellitus, UAT	8	
Hyperlipidemia, UAT	14	
Moderate impact heart-related conditions	18	44
Prior myocardial infarction (>1 year)	11	27
Valve disease	7	17
High impact heart-related conditions	23	56
Chronic, multivessel coronary disease, UAT	19	46
Atrial fibrillation, UAT	9	22
Conduction disturbances, UAT	10	24
Hypertensive heart disease, UAT	4	10
Beta-blockers and/or ACEI/ARB background treatment	27	65
Age-adjusted Charlson Comorbidity Index		
Median	8	
Range	5–12	

ULN, upper limit of normal range; ECOG, Eastern Cooperative Oncology Group; GFR, glomerular filtration rate; UAT, under active treatment; LVEF, left ventricular ejection fraction; IPI, international prognostic index; ACEI, angiotensin-converting-enzyme inhibitors; ARB, angiotensin receptor blockers.

The distribution of heart-related conditions with ‘life threat’ impact is also detailed in Table I. Moderate impact conditions, such as prior myocardial infarction and valve disease were present in 11 and seven cases respectively, while a large proportion of patients (56%) presented high-impact situations mainly consisting of ischaemic and arrhythmic cardiac diseases under active treatment (Table I). Cardiac risk factor hypertension, diabetes mellitus and dyslipidaemia were under medication in 34 cases, while moderate chronic renal failure (glomerular filtration rate 30–50 ml/min) affected 12% of patients. Two-thirds of patients had background treatment including ACEI/ARB and/or beta blockers. Median basal echocardiographic LVEF for the entire study population was 57% (range,45–67). Median aaCCI was 8 (range, 5–12).

### Primary endpoints

The scheduled six courses of R-COMP-14 were completed by 27 patients (67%), including two patients who received two additional courses as CR consolidation for a destructive bone involvement (Table II). In these latter patients, aged 72 and 81 years, radiotherapy was excluded to allow osteosynthesis (right omerus, epiphyseal-metaphyseal area) in one case, and avoid post-actinic damage (left maxillary sinus, pterigoides laminae, left greater sphenoid wing and ethmoid septum) in the other. Three patients, aged 82, 69 and 78 years, all in CR at intermediate restaging, were considered to have completed their treatment after five courses (Table II): the first patient did not attend the scheduled sixth course for logistic reasons after relatives referred her to a distant community dwelling; the second skipped his sixth course so as not to delay radiofrequency ablation treatment of hepatocellular carcinoma; in the third patient, a psychiatric counsellor, upon the occurrence of a severe isolated memory loss of unknown cause, advised skipping the last course. These three complete responders were not considered as failures due to toxicity, treatment discontinuation or refusal.

**Table II:** Feasibility of R-COMP-14: status of patients and cause of termination of therapy after each cycle

	No. of patients (%)								
	
	Cycle 1	Cycle 2	Cycle 3	Cycle 4	Cycle 5	Cycle 6	Cycle 7	Cycle 8	Total
Patients receiving therapy	41(100)	39 (95)	36 (88)	31 (80)	30 (73)	27(67)	2	2	
Early death	2	1							3 (7)
Discontinuation for toxicity		2	3	1					6 (15)
LVEF <40%			2	1					3 (7)
CTCAE Grade 3 arrhythmia		1							2 (5)
CTCAE Grade 4 arrhythmia			1						
Disseminated Zoster		1							1
Diversion for no response				2					2
Treatment completed					3	25		2	30 (73)

LVEF, left ventricular ejection fraction; CTCAE, Common Terminology Criteria for Adverse Events version 3.0.

Overall, a total of 208 courses were delivered, with a median time to recycle of 15·6 d (range, 13–29). Treatment delivery achieved an average RDI of 88·6% (cyclophosphamide 86%, NPLD 89%, vincristine 91%). Three patients received CNS prophylaxis with intrathecal liposomal cytarabine due to epidural (*n*=2) and paranasal sinus (*n*=1) localizations.

Discontinuation for toxicity occurred in a total of six patients (15%). This was due to widespread herpes zoster infection (*n*=1) and grade 3 cardiac events (*n*=5), namely decrease in LVEF (*n*=3) and grade 3 and grade 4 arrhythmia (*n*=2) (Table II). After stopping R-COMP-14, all these six patients did not receive any further chemotherapy but were given additional doses of rituximab, at biweekly intervals, up to a total of eight (*n* = 4) or ibritumomab-tiuxetan (*n* = 2). The cumulative incidence of failures due to early and late cardiac events was 17% (7/41; 95% CI 8% to 31%).

The ORR was 73% with 28 patients achieving CR (68%; 95% CI 54% to 82%). After a median period of observation for TTF of 27 months, 20 failures according to the protocol were observed: three early therapy-related deaths [at day +12 (undefined cause) and +13 (sepsis) of the first course, and at day +18 (sepsis) from the second cycle, respectively], eight treatment discontinuations [no response, i.e. less than PR/progressive disease (*n* = 2), toxicity (*n* = 6)], six relapses, three lymphoma-unrelated deaths in continuous CR, due to decompensated cirrhosis (*n* = 1) and late cardiac events (*n* = 2).

**Fig 1:**
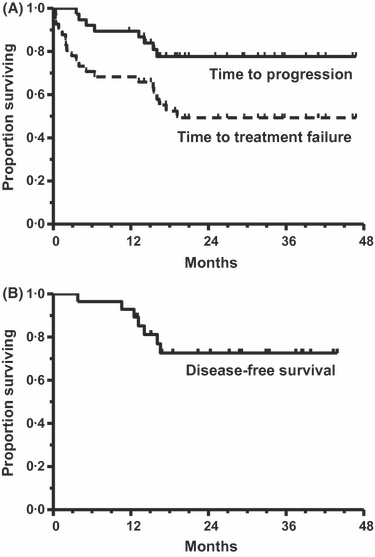
Kaplan–Meier survival curves for the whole cohort (*n* = 41) (A) and complete responders (*n* = 28) (B).

**Fig 2:**
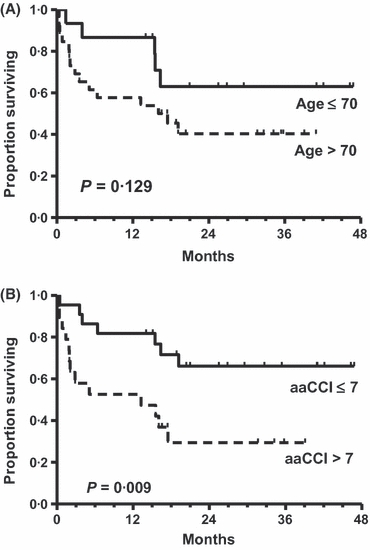
Time to treatment failure according to age cut-off 70 years (A) and aaCCI (B).

**Fig 3:**
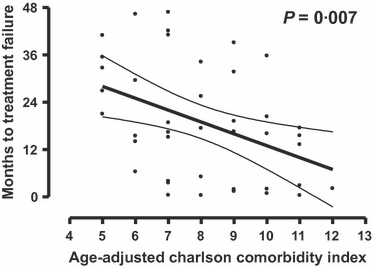
Correlation of aaCCI with treatment failure. Regression line with 95% confidence band.

The 4-year TTF was 49% (95% CI 33% to 65%, median 19·2 months) without a significant advantage for patients younger than 70 years (63% vs. 40%; *P* = 0·12) (Table III, Figs 1A and 2A). In contrast, a statistically significant correlation with treatment failure was found for the aaCCI (*r*^2^=0·17, *P* = 0·007) (Fig 3). In particular, patients with an aaCCI score ≤7 (66%; 95% CI 46–86) displayed a longer TTF as compared to those with a score >7 (29%; 95% CI 8% to 49%)(*P* = 0·009) (Fig 2B). Early deaths and toxicity-related therapy discontinuations were also significantly more frequent among patients with aaCCI scores >7 (*P* = 0·02), while these events were not predicted by chronological age (Table III).

**Table III:** Response to therapy and events according to age and aaCCI

	Total			Age ≤ 70 years		Age > 70 years			aaCCI ≤ 7		aaCCI > 7		
					
	(*n*=41)			(*n*=15)		(*n*=26)			(*n*=19<)		(*n*=22)		
					
	*n*	%	95% CI	*n*	%	*n*	%	P*	*n*	%	*n*	*%*	P*
Overall response rate	30	73	60–87	13	87	17	65	0·16	17	89	13	59	0·47
CR	28	68	54–82	12	80	16	61	0·30	15	79	13	59	0·62
PR	2			1		1			2				
Less than PR	2			1		1			1		1		
Discontinuation	6	15	4–25	0		6	23}	0·11	1		5	23}	0·02
Early therapy-related deaths	3	7	1–15		1	2					3		
Relapsed	6	21	6–37										
Deaths for lymphoma	6	15	4–25	2		4			4		2		
Deaths unrelated	1			1							1		
Late therapy-related deaths	2			1		1			1		1		
Alive	29	70	57–85	11	73	18	69		14	73	15	68	
4-year survival													
TTF		49	33–65		63		40	0·12		66		29	0·009
TTP		77	64–91		84		67	0·32		77		70	0·88
OS		67	52–83		70		64	0·75		71		65	0·49
DFS		72	55–90		80		66	0·40		77		67	0·63

aaCCI, age-adjusted Charlson comorbidity index; CR, complete response; PR, partial response; TTF, time to treatment failure; TTP, time to progression; OS, overall survival; DFS, disease-free survival.

* All *P* values are two-sided.

**Fig 4:**
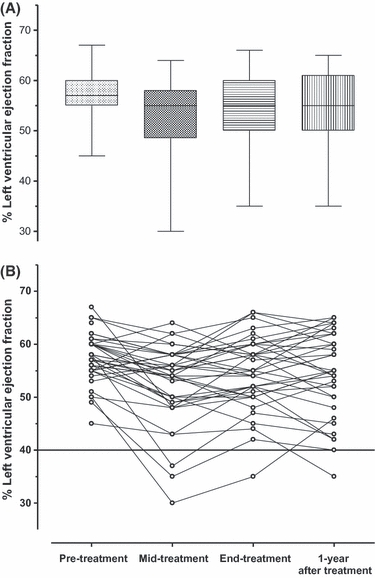
Variations in left ventricular ejection fraction (LVEF) throughout treatment up to 1-year after completion of treatment expressed in quartiles (A) and individual values (B). The bold line indicates the limit below which grade 3 and 4 toxicity for ‘left ventricular systolic dysfunction’ occur.

Median values of LVEF for the entire study population documented at 1 year after completion of treatment were superimposable to those present before treatment (Fig 4A), but differences in quartiles were evident. At midtreatment evaluation the LVEF had a 5% to 10% decrease in eight patients, while a >10% reduction was observed in five others. Among the latter group, three patients had their therapy stopped per protocol becuase the CTCAE limit for grade 3 toxicity, i.e. a LVEF <40%, was reached. Nevertheless, the remission status achieved by these three patients allowed a time to progression of 12+, 15+ and 16+ months, which was paralleled by a progressive recovery of their LVEF values. At 1 year from completion of treatment, 23 of 29 surviving patients did not show significant differences in LVEF as compared to pre-therapy values. A 10% to 15% reduction of LVEF was recorded in six cases, while two patients displayed a LVEF value below 40% (Table II and Fig 4B).

Haematological toxicity was acceptable, with grade 3 and 4 thrombocytopenia occurring in nine patients (22%), grade 3 and 4 anaemia in six (15%) and grade 3 and 4 infections in eight (19%). Notably, at the time of final restaging, focal lung parenchymal uptake at FDG-PET was found in six patients (five of them were aged >75 years), which qualified as pulmonary infection (no *Pneumocystis spp*) and was treated effectively with antibacterial agents.

### Secondary endpoints

Among 28 complete responders, eight events were recorded, namely five relapses and three deaths in CR at months 13, 14 and 16, respectively; the first death was due to hepatic cirrhosis, the second to a sudden death, considered per protocol as a ‘cardiac event’ in the absence of any documented aetiology, the third to CHF. According to the adopted standardized criteria for DFS endpoint (Cheson *et al*, 2007), the first patient was censored at month 13, while the second and the third cases were both included as events, with a 4-year DFS of 72% (95% CI 55% to 90%) (Fig 1B).

Overall, 12 patients (29%) died (Table III), yielding to an estimated 4-year OS of 67% (95% CI 52% to 83%). Causes of death included early toxic event (*n* = 3), lymphoma progression (*n* = 6) and lymphoma-unrelated cause (*n* = 3). The TTP rate was 77% (95% CI 69% to 91%) (Table III and Fig 1A). TTP survival analysis also included the six patients who had discontinued treatment due to toxicity. Among them, there were four CRs with a TTP of 12, 16, 20+ and 29+ months respectively, and two initial PRs whose TTP ended at months 8 and 14.

## Discussion

This prospective study addressed two issues relevant to the management of DLBCL in the elderly. The first was to assess whether substitution of NPLD for conventional doxorubicin allowed the safe application of a dose-densified R-CHOP-like regimen to older patients with poor-risk disease (IPI ≥ 3) and moderate to high ‘life threat’ impact (NIA/NCI) cardiac comorbidity. The second was to evaluate the potential clinical benefit of R-COMP-14 in terms of CR, TTF and DFS rates in this subset of high-risk patients.

About two-thirds of patients completed the entire six-course programme with a median average RDI approaching 90% for all of the drugs. These proportions are valuable because about one-third of patients were older than 75 years, i.e. above the upper age limit for enrollment into baseline studies (Feugier *et al*, 2005; Pfreundschuh *et al*, 2004). Response rate was aligned with results of non-cardiopathic patients with unfavourable IPI profiles (Pfreundschuh *et al*, 2008). The substantial DFS achieved supports the use of dose-dense treatment is worth ensuing also when age and cardiac comorbidity increase the complexity of lymphoma management and may affect survival (Aapro *et al*, 2010). The TTF, though hardened by a strict definition of failures, was also in keeping with recent literature (Feugier *et al*, 2005; Pfreundschuh *et al*, 2008). Early treatment discontinuation for toxicity occurred in only 15% of patients, all older than 70 years. All of these patients, despite receiving a reduced number of R-COMP-14 courses (median 3, range 2–4), achieved objective responses and contributed to the TTP curve. This indicates that delivery of even fewer cycles of doxorubicin-based chemotherapy remains an option to pursue in non-frail patients older than 70 years (Chrischilles *et al*, 2003). In this regard, application of fewer courses of densified R-COMP-14 might be of value.

Although the importance of dose-intensity in aggressive NHL was confirmed in the rituximab era (Hirakawa *et al*, 2010), the superiority of R-CHOP-14 over R-CHOP-21 is highly debated, also due to interim results of ongoing randomized trials (Cunningham *et al*, 2009; Delarue *et al*, 2009). While these studies will eventually clarify the issue, R-CHOP-14 remains a possible option for elderly patients with a high IPI risk, given the significant relapse rate of R-CHOP-21 in this specific patient population (Feugier *et al*, 2005) and the inadequate results of salvage treatments (Corazzelli *et al*, 2009; Gisselbrecht *et al*, 2009). Concerns about the toxicity of biweekly R-CHOP, especially with regard to cardiac morbidity and infectious risk (Johnson, 2006; Tadmor *et al*, 2010), may restrain physicians from proposing this strategy to elderly individuals. Irrespective of dose-densification, older DLBCL patients with comorbidity, underlying heart disease, and/or poor PS are often precluded ‘*a priori*’ from a curative treatment. These conditions should not ‘*per se*’ contraindicate the application of R-CHOP (Johnson, 2006). This may reflect a cultural reluctance to treat individuals with a presumed defective resilience to treatment toxicity and the inadequacy of instruments to properly identify subclinical damage and prompt timely preventive measures and/or treatment interruptions (Aapro *et al*, 2010; Thieblemont & Coiffier, 2007). The present study was designed to overcome this conceptual framework by implementing a dose-dense anthracycline-containing regimen for patients with cardiac comorbidity through strict patient monitoring.

The cumulative incidence of failures due to grade 3–5 cardiac events was only 17% in our study. This proportion is apparently higher than that reported (5%) in a study of 72 elderly DLBCL patients treated with a 3-weekly R-COMP regimen (Luminari *et al*, 2010). However, strict enrollment criteria limited the overall burden of comorbidity in this latter study, as witnessed by the presence of clinically significant arrhythmic or ischaemic disease in only 14% of patients and the absence of cardiovascular risk factors in 47% of the cases (Luminari *et al*, 2010). A 5% to 15% incidence of grade 3–5 cardiovascular events was reported in two small series of elderly patients treated with an R-COMP-21 regimen (Rigacci *et al*, 2007; Visani *et al*, 2008). However, concomitant accrual of naïve and pretreated or frail patients, coupled to the heterogeneity and variable severity of cardiac comorbidity and cardiovascular risk factors in these two series, hampers a comparison with our study.

In five of our patients with normal pretreatment LVEF values, grade 3 cardiac events with LVEF reduction occurred between the 2nd and 4th course of R-COMP-14, despite the low cumulative dose of NPLD. Previous CHOP studies have described LVEF declines at a cumulative doxorubicin dose of only 200 mg/m^2^ (Limat *et al*, 2003; Nousiainen *et al*, 2002), highlighting diastolic dysfunction and heart failure with preserved left ventricular systolic function as early predictors of cardiotoxicity (Pudil *et al*, 2008). This suggests that LVEF should not be the only tool for the assessment of early cardiotoxicity in older NHL patients with preexisting cardiopathy but that additional cardiological evaluations and biomarkers studies are necessary (Albini *et al*, 2010; Altena *et al*, 2009). Serial measurements of proBNP levels were available in 44% of our patients (data not shown), but the presence of pre-treatment proBNP levels above the age-adjusted cut-off values, and their variations due to cardiological therapy, anaemia, infections and renal disease, affected the specificity and predictive value of this biomarker, as previously reported (Dodos *et al*, 2008).

The aaCCI, a prototypical and reliable comorbidity index, which includes lymphoma among the weighted medical conditions, was a robust predictor of treatment outcomes and survival in our cohort of cardiopathic DLBCL patients. Intriguingly, aaCCI scoring was a better predictor for early death and toxicity-related therapy discontinuation than age itself. This data supports the application of indexes, such as the aaCCI, in the specific situation of elderly NHL patients with heart disease. In this context, comorbidity assessment may account for breakthrough non-cardiac morbidities, including chronic renal failure, lung diseases, endocrine disorders (Khan *et al*, 1999), neutropenic sepsis (Ammann *et al*, 2001; ver Elst *et al*, 2000) or hypovolemic/hypotensive problems (Arlati *et al*, 2000), which can trigger secondary cardiac dysfunctions unrelated to cardiotoxicity from chemotherapy.

Patients in our study could have benefited from a protective effect and/or improved cardiac compensatory mechanisms from their background cardiological therapy. As it is unclear whether preexisting heart disease increases susceptibility to anthracycline-induced damage or the impaired cardiac functional reserve increases vulnerability of older patients to additional myocardial injures (Hershman *et al*, 2008), a beneficial pharmacological action from beta-blockers and/or ACEI or ARB assumed by about two-thirds of our patients may be supposed. Notably, while a large amount of data on cardioprotective strategies was generated in childhood cancers and solid tumours (Cardinale *et al*, 2006; Lipshultz & Colan, 2004), information is scanty for DLBCL, a disease that typically affects elderly individuals and is exquisitely sensitive to anthracyclines. Therefore, future studies on treatment of old and very old patients with aggressive NHL should be designed to evaluate, together with less cardiotoxic anthracycline formulations, cardioprotective strategies and agents as well as noninvasive monitoring markers and methods to detect early signs of cardiotoxicity (Albini *et al*, 2010; Altena *et al*, 2009).

We documented the feasibility and efficacy of an R-COMP-14 regimen in poor-risk elderly DLBCL patients with significant cardiac comorbidity. Many of these high-risk patients, who eventually achieved complete and durable responses, would probably have been denied curative treatment based on the comorbidity burden and age. In the specific setting of cardiopathic patients with aggressive NHL, value of liposomal doxorubicin deserves comparison with the less cardiotoxic protracted infusions of doxorubicin or newer anthracycline congeners.

## Supporting Information

**Table S1.** Patient characteristics and response according to Stage of accrual in the study.
