# Supplementary material for: Biweekly rituximab, cyclophosphamide, vincristine, non-pegylated liposome-encapsulated doxorubicin and prednisone (R-COMP-14) in elderly patients with poor-risk diffuse large B-cell lymphoma and moderate to high ‘life threat’ impact cardiopathy
Source: Br J Haematol. 2011 Sep;154(5):579–89. doi: 10.1111/j.1365-2141.2011.08786.x (PMC3258483; doi:10.1111/j.1365-2141.2011.08786.x)
Supplement: Supplementary file 1 [file bjh0154-0579-SD1.doc]

**Table S1**. Patient characteristics and response according to Stage of accrual in the study

|  | | Stage-one | |  | Stage-two |  |
| --- | --- | --- | --- | --- | --- | --- |
| Characteristics* | | *n* | % |  | *n* | % |
| Patient entered | | 19 | 100 |  | 22 | 100 |
|  | |  |  |  |  |  |
| Age at treatment (years) | |  |  |  |  |  |
|  | median ( range) | 74 (63-82) | |  | 73 (62-82) | |
|  | 61-70 | 7 | 37 |  | 8 | 36 |
|  | 71-80 | 10 | 53 |  | 11 | 50 |
|  | > 80 | 2 | 10 |  | 3 | 14 |
| Male gender | | 10 | 53 |  | 13 | 59 |
| Clinical Stage | |  |  |  |  |  |
|  | II | 3 | 16 |  | 1 | 5 |
|  | III | 4 | 21 |  | 6 | 27 |
|  | IV | 12 | 63 |  | 15 | 68 |
| B symptoms | | 10 | 53 |  | 10 | 45 |
| No of extranodal sites ≥2 | | 5 | 26 |  | 9 | 41 |
| Bone marrow involvement | | 3 | 16 |  | 6 | 27 |
| Lactate dehydrogenase > ULN | | 14 | 74 |  | 19 | 86 |
| ECOG Performance Status ≥2 | | 6 | 31 |  | 7 | 32 |
| Standard IPI score | |  |  |  |  |  |
|  | 3 | 12 | 63 |  | 12 | 54 |
|  | 4-5 | 7 | 37 |  | 10 | 46 |
| Basal LVEF value (%) | |  |  |  |  |  |
|  | median | 58 |  |  | 57 |  |
|  | range | 45-65 |  |  | 49-67 |  |
| Cardiovascular risk factors | |  |  |  |  |  |
|  | Chronic renal failure (GFR 30-50 ml/min/1.73 m^2^) | 2 |  |  | 3 |  |
|  | Hypertension, UAT | 5 |  |  | 7 |  |
|  | Diabetes mellitus, UAT | 3 |  |  | 5 |  |
|  | Hyperlipidemia, UAT | 6 |  |  | 8 |  |
| Moderate impact heart-related conditions | | 8 | 42 |  | 10 | 46 |
| High impact heart-related conditions | | 8 | 42 |  | 13 | 59 |
| Beta-blockers and/or ACEI/ARB background treatment | | 11 | 58 |  | 16 | 73 |
| Age-adjusted Charlson Comorbidity Index | |  |  |  |  |  |
|  | median | 7 |  |  | 8 |  |
|  | Range | 5-12 |  |  | 5-12 |  |
| Overall Response Rate | | 14 | 74 |  | 16 | 73 |
|  | CR | 13 | 68 |  | 15 | 68 |
|  | PR | 1 |  |  | 1 |  |
| Less than PR | | 1 |  |  | 1 |  |
| Early death | | 1 |  |  | 2 |  |
|  | Sepsis | 1 |  |  | 1 |  |
|  | Undefined |  |  |  | 1 |  |
| Discontinuation for toxicity | | 3 | 16 |  | 3 | 14 |
|  | Cardiac event | 2 |  |  | 3 |  |
|  | Infection | 1 |  |  |  |  |
|  |  |  |  |  |  |  |

ULN, upper limit of normal range; GFR, glomerular filtration rate; UAT, under active treatment; LVEF, left ventricular ejection fraction; IPI, international prognostic index; ACEI, angiotensin-converting-enzyme inhibitors; ARB, angiotensin receptor blockers; CR, complete response; PR, partial response.

* All differences between characteristics of patients accrued in Stage-one and Stage-two were not statistically significant at univariate analysis (see material and methods).
